# An intelligent stochastic optimization approach for air cargo order allocation under carbon emission constraints

**DOI:** 10.1371/journal.pone.0319973

**Published:** 2025-04-10

**Authors:** Zhenzhong Zhang, Ling Zhang, Deqiang Fu, Weichun Li

**Affiliations:** 1 CAAC Academy, Civil Aviation Flight University of China, Chengdu, Sichuan, China; 2 School of Modern Posts, Chongqing University of Posts and Telecommunications, Chongqing, China; Cyprus International University Faculty of Engineering: Uluslararasi Kibris Universitesi Muhendislik Fakultesi, TÜRKIYE

## Abstract

In air cargo transportation, effective order allocation is crucial for improving the efficiency of business operations and reducing environmental impact. In this paper, we study a high-dimensional stochastic order allocation problem that assigns uncertain orders to different types of aircraft for transportation. Considering the carbon emission and the uncertainty of customer order arrivals in the actual transportation environment, a stochastic optimization model considering the cost of carbon emission is established with the objective of maximizing the expected profit from order transportation. A new intelligent optimization method is introduced for addressing the order assignment problem under carbon emission constraints by combining the improved adaptive large-scale neighborhood search algorithm with the scenario generation technique. The method finds the optimal solution through an improved adaptive large-scale neighborhood search algorithm and uses a scenario generation technique to generate the scenarios required for evaluating candidate solutions to the high-dimensional stochastic optimization problem. Experimental results show that this method surpasses the compared optimization methods regarding both optimization capability and optimization efficiency.

## 1. Introduction

The assignment problem of air cargo transportation orders is a complex optimization issue involving the assignment of *m* orders to *n* aircraft, where each aircraft has a different transportation capacity. The objective is to ensure that each order is assigned to only one aircraft and that all orders are transported appropriately for optimal productivity [1]. This problem is a generalized assignment problem (GAP). GAP has a wide range of applications in several fields, including computer network analysis [2], transportation planning [3], and production planning [4]. The air cargo industry is a vital component of global logistics, providing indispensable support to modern supply chains with its fast and efficient transportation capabilities. However, as the demand for air cargo continues to grow, the industry faces major challenges in controlling transportation costs and reducing carbon emissions while meeting customer needs. In air cargo operations, the order assignment problem is particularly critical, as it directly impacts aircraft load efficiency, transportation time, and environmental effects. Therefore, optimization research on the order assignment problem in air cargo has significant theoretical and practical importance.

Most prior researches on the order assignment problem rely on deterministic assumptions, such as preset transportation times and fixed order demands [5,6]. In the real-world air transportation environment, various uncertainties frequently occur, including customer order cancellations [7] and unpredictable transportation times [8]. Deterministic assumptions do not meet the practical needs, and the solutions obtained based on deterministic assumptions make it difficult to achieve the optimized objectives in real production environments due to various inherent uncertainties, as stated by Liao et al. [9]. The deterministic assumptions are not consistent with the practical needs. Solutions derived under these assumptions can be highly misleading and may result in cost increases exceeding 50% compared to those obtained in uncertain environments [10].

The existing literature on GAP has primarily concentrated on deterministic problems [11]. The research exploring GAP with stochastic parameters is very limited, as is the research on the stochastic generalized assignment problem (SGAP) [12]. In 2000, Albareda and Fern introduced a heuristic approach to solving the stochastic assignment problem with boundary constrained [13]. Dyer and Frieze provide a case where resource consumption coefficients and cost coefficients are uniformly distributed [14]. These researchers devised enumeration algorithms with polynomial-time complexity using linear relaxation, yet they are ineffective for solving stochastic GAP problems on a large scale. Albareda-Sambola et al. modelled job demand as a stochastic variable and proposed a two-stage stochastic planning method. jobs are first allocated to the machines, and then the allocation scheme is adjusted after the demand is determined, and overloaded jobs on the machine need to be reassigned and penalties incurred [15]. Albareda and Fern devised an algorithm to address this SGAP, however, its applicability is limited [14]. Albareda et al. employed the branch and cut method to address SGAP, which involves 4-15 operations [15].

Real-world uncertainty can be modelled using various forms, including rough and fuzzy variables [16]. Midya and Roy developed an efficient method for solving problems by utilizing rough or fuzzy variables to capture unpredictable elements [17]. In studies of demand uncertainty, these uncertainties are typically modelled using random variables [18]. This randomness has been utilized in various issues, including the randomized route problem [19]. Sarin and Sherali modelled the operating time as a random variable and proposed a branch and price-based approach for solving the issue, which was shown to outperform the branch-and-cut method [12], and the branch and price combined with random sampling (RS) are more likely to obtain a near-optimal solution and save over ten times the CPU time in dealing with larger-scale problem involving 10-22 orders with dimensions ranging from 2^5^ to 2^11^ [15].

Although RS-based methods have shown effectiveness in solving high-dimensional stochastic GAPs, they often require many scenarios to represent the characteristics of the random variables, leading to computationally intensive and slow convergence of RS-based methods. Few studies have proposed more efficient methods for solving stochastic GAPs containing high-dimensional random variables. The stochastic GAP considered in the study is focused on maximizing the anticipated revenue of order transportation by selecting appropriate customer orders and assigning them to appropriate aircraft. In this case, the profit of order transportation is the difference between the transportation revenue and the cost of transportation and the cost of excess carbon emission penalties. In this problem, the probability of selecting an order for transportation is known, but the uncertainty of order arrival leads to a high dimensional random variable, and how to effectively deal with such a high dimensional stochastic variable is a significant challenge in the realm of optimization problems.

GAPs such as order allocation are NP-hard combinatorial optimization problems. To solve GAP, researchers have proposed numerous methods, mainly including exact algorithms [20,21] and heuristic algorithms [4,22]. Although exact algorithms can find optimal solutions, they are often infeasible in practical applications due to excessive computational time. In contrast, heuristic and intelligent optimization algorithms show effectiveness in solving GAP. The introduction of stochasticity makes the GAP more complex. Traditional RS methods require many scenarios to simulate random features when dealing with high-dimensional random variables, resulting in long computation time. Therefore, a scenario generation (SG) technique based on iterative selection of scenarios can be used, which evaluates candidate solutions by identifying scenarios that lead to penalty costs and those that do not.

Employing efficient optimization algorithms to identify promising solutions is essential for enhancing optimization efficiency when tackling high-dimensional stochastic optimization problems. A very large-scale neighborhood search (VLNS) algorithm is considered to have great potential in improving optimization efficiency [23]. Adaptive large neighborhood search (ALNS) is an effective algorithm for order assignment issues and outperforms simulated annealing algorithms, genetic algorithms, tabu search algorithms, ant colony optimization algorithms, etc [24]. This paper introduces a modified ALNS algorithm, known as MALNS, which enhances optimization efficiency through the integration of a probability-based selection mechanism with ALNS in Chen et al. [25].

This study proposes an intelligent stochastic optimization method that merges the optimization capability of the MALNS with the effectiveness of the SG technique for handling high-dimensional random variables. The proposed methodology is particularly suitable for complex, high-dimensional stochastic optimization problems, such as air cargo order allocation with uncertain demands and environmental constraints. Its unique combination of scenario generation techniques and adaptive large-neighborhood search ensures both computational efficiency and solution quality, making it highly applicable to real-world logistics and transportation problems. This paper makes two primary contributions. Firstly, it explores a high-dimensional stochastic order allocation problem in air cargo transportation that takes carbon emissions into account. Secondly, it proposes an effective stochastic optimization method that combines a MALNS algorithm with the SG technique. The MALNS algorithm is developed to enhance optimization efficiency, while the SG technique, based on iterative scenario selection, is integrated to manage high-dimensional uncertainties.

This paper is structured as follows. The stochastic order allocation problem and its mathematical model are introduced in Section 2. An intelligent optimization method that combines SG and MALNS is proposed in Section 3. Numerical experiments are carried out and findings are discussed in Section 4. Finally, this paper is summarized in Section 5.

## 2. Problem description and modeling

### 2.1. Description of the problem

In this paper, we study the problem of allocating *n* orders (represented as *i* and $i\in N=\left\{1,\cdot \cdot \cdot ,n\right\}$) to *m* aircraft (represented as *j* and $j\in M=\left\{1,2,\cdot \cdot \cdot ,m\right\}$) in a transportation setting characterized by order uncertainty. All orders can be processed at moment 0. An order cannot be interrupted once the transportation has started until the transportation is completed. The cost and time for order *i* to be transported by aircraft *j* are $c_{ij}$ and $t_{ij}$. Order *i* has a delivery date of $d_{i}$. Delays in delivery result in a penalty cost, calculated using a factor of *δ*. Order uncertainty implies that a customer may cancel order *i* where $p_{i}$ represents the probability of selecting order *i* for transportation. The problem aims to optimize the expected revenue from transporting orders.

The optimization problem of order assignment in air cargo transportation is prevalent in the daily operations of air cargo companies. Specifically, in real-world air cargo environments, customer order arrivals are uncertain, while aircraft capacity (including weight and volume) and transportation time are strictly limited. Moreover, aircraft carbon emissions are closely related to their payload, and exceeding the carbon emission allowance results in substantial penalties.

If order *i* is chosen to be transported, $x_{i}=1$; otherwise $x_{i}=0$. If order *i* is assigned to aircraft *j*, $x_{ij}=1$; otherwise $x_{ij}=0$. The maximum loaded weight and volume for aircraft *j* are $W_{j}$ and $V_{j}$, and the weight and volume for order *i* are $o_{i}^{w}$ and $o_{i}^{v}$, respectively. The carbon emissions of an aircraft are linearly related to the weight of the cargo with a correlation coefficient of *γ*. The total transportation carbon emissions from aircraft transportation orders may exceed their carbon allowances. Let $\alpha_{j}$ denote the rated carbon emissions of the aircraft *j*. If the total carbon emissions from aircraft *j* exceed $\alpha_{j}$, an overage penalty is incurred with a factor of *ε*.

In an uncertain transportation environment, there are multiple order demand scenarios. A scenario set *G* is set where each scenario s∈G represents a particular combination of order demand. For any of the scenarios *s*, if an order *i* occurs, i.e., a demand exists for the ith order under scenario *s*, then $y_{i}^{s}=1$; otherwise $y_{i}^{s}=0$. When evaluating a potential order allocation scenario, if demand exists for order *i* under a given scenario ($y_{i}^{s}=1$), but the order is not considered in the allocation scheme ($x_{i}=0$), or if demand does not exist for order *i* ($y_{i}^{s}=0$) but is included in the allocation scheme ($x_{i}=1$), then the order will not be selected for transportation. The probability of scenario *s* is denoted by $\pi^{s}$, and in the case where all the occurrences of the order are independent of each other, $\pi^{s}$ can be calculated as follows:


$$\pi^{s}=\underset{i}{\Pi }\left(y_{i}^{s}\cdot p_{i}+\left(1-y_{i}^{s}\right)\left(1-p_{i}\right)\right)$$ (1)

For each order *i*, the start shipping time is defined as $b_{i}$, the completion shipping time is defined as $f_{i}$, and the revenue obtained by transporting order *i* is $v_{i}$. The value of $e_{j}^{s}$ is used to indicate the total carbon emissions of aircraft *j* under the scenario *s* that exceeds its rated carbon emissions, and $e_{j}^{s}$ is equal to 0 if the total carbon emissions of aircraft *j* is less than the rated carbon emissions. Additionally, $x_{ij}^{s}$ is used to indicate whether the order *i* is scheduled for transported on aircraft *j* in scenario *s*, if yes $x_{ij}^{s}=1$, otherwise $x_{ij}^{s}=0$.

### 2.2. Mathematical modeling


$$\underset{x\in Χ}{\max}\sum_{s\in G}\pi^{s}\left(\sum_{i\in N}\sum_{j\in M}\left(y_{i}^{s}\cdot x_{i}\cdot \left(\left(v_{i}-c_{ij}\cdot x_{ij}\right)+\delta \cdot \left(f_{i}-d_{i}\right)\right)\right)-\sum_{j\in M}\left(\varepsilon \cdot e_{j}^{s}\right)\right)$$ (2)

s.t.


$$e_{j}^{s}=\max\left(\sum_{i\in N}(\gamma \cdot y_{i}^{s}\cdot x_{ij}\cdot o_{i}^{w})-a_{j},0\right)�\forall j,s$$ (3)

$$f_{i}=b_{i}+\sum_{j=1}^{m}\left(x_{ij}\cdot t_{ij}\right),\forall i$$ (4)

$$\sum_{i=1}^{n}\left(o_{i}^{w}\cdot x_{ij}\right)\leq W_{j},\;\forall j$$ (5)

$$\sum_{i=1}^{n}\left(o_{i}^{v}\cdot x_{ij}\right)\leq V_{j},\;\forall j$$ (6)

$$\sum_{j=1}^{m}x_{ij}^{s}=1,\;\forall i,x_{i}y_{i}^{s}=1$$ (7)

$$x_{i}\text{=}\sum_{j}^{m}x_{ij},\;\forall i$$ (8)

$$\sum_{j=1}^{m}x_{ij}^{s}=0,\;\forall i,x_{i}y_{i}^{s}=0$$ (9)

$$x_{i}\in \{0,1\},\;\forall i$$ (10)

$$x_{ij}^{s}\in \{0,1\},\;\forall i,j,s$$ (11)

$$y_{i}^{s}\in \{0,1\},\;\forall i,s$$ (12)

$$x_{ij}\in \{0,1\},\;\forall i,j$$ (13)

Formula (2) is designed to calculate the anticipated revenue on transportation orders, where $\sum_{i\in N}\sum_{j\in M}\left(y_{i}^{s}\cdot x_{i}\cdot \left(\left(v_{i}-c_{ij}\cdot x_{ij}\right)+\delta \cdot \left(f_{i}-d_{i}\right)\right)\right)-\sum_{j\in M}\left(\varepsilon \cdot e_{j}^{s}\right)$ represents the difference between the revenue from transportation orders under scenario *s* and the cost of transportation and penalty costs. Equation (3) calculates the value by which the total carbon emissions for aircraft *j* under scenario *s* exceeds its carbon allowance. Equation (4) is used to calculate the transportation completion time for the order *i*. Equations (5) and (6) indicate that the weight and volume of orders transported by aircraft do not exceed their normal loading capacity. Formulas (7) and (8) guarantee that each order is assigned to exactly one aircraft for transportation. Constraint (9) states that if there is no demand for order *i* in a scenario or if the candidate solution does not consider this order, then the order will not be selected for transportation. Equations (10)–(13) specify the range of values for each variable.

When scenarios are sufficiently numerous, $\sum_{s\in S}\pi^{s}y_{i}^{s}$ tends to the marginal probability that an order *i* is not canceled $p_{i}$. Thus, Equation (2) can be restated as follows.


$$\underset{x\in Χ}{\max}\left(\sum_{i\in N}\sum_{j\in M}\left(p_{i}\cdot x_{i}\cdot \left(\left(v_{i}-c_{ij}\cdot x_{ij}\right)+\delta \cdot \left(f_{i}-d_{i}\right)\right)\right)-\sum_{s\in G}\sum_{j\in M}\left(\varepsilon \cdot \pi^{s}\cdot e_{j}^{s}\right)\right)$$ (14)

In Equation (14), $\sum_{i\in N}\sum_{j\in M}\left(p_{i}\cdot x_{i}\cdot \left(\left(v_{i}-c_{ij}\cdot x_{ij}\right)+\delta \cdot \left(f_{i}-d_{i}\right)\right)\right)$ can be calculated directly. $\sum_{s\in G}\sum_{j\in M}\left(\pi^{s}e_{j}^{s}\right)$ can be calculated using the method in Section 3.3.

## 3. Intelligent stochastic optimization method

### 3.1. Overview of the methodology

This paper introduces a novel intelligent optimization approach, named scenario-guided intelligent neighborhood optimization (SGINO), to address the complex stochastic order allocation problem in air cargo while taking carbon emissions into account. The method utilizes the MALNS optimization strategy, which resembles the ALNS but introduces the order selection mechanism based on probability in VLNS to improve the efficiency of searching for neighborhood solutions. The *τ* -neighborhood solution is defined as the solution obtained by selecting *τ* (2≤τ≤n) orders from the individual and then reassigning them using the order insertion approach in Section 3.4. The application of the selection mechanism to choose high-value solutions in the neighborhood solutions leads to a substantial reduction in the time required for solution evaluation. The SG technique enhances the efficiency of solution evaluation by dividing the evaluation scenarios of a candidate solution into two parts.

Algorithm 1 shows the pseudo-code of the stochastic optimization algorithm proposed in this paper. Line 1 sets the initial parameters of the algorithm. Line 2 generates the initial solution using the method in Section 3.2, with the basic principle of assigning the order to the plane capable of completing the transportation first. Line 3 evaluates the initial solution’s fitness using the SG technique. Line 4 checks that the algorithm satisfies the termination condition. If the termination condition is not met, continue with lines 5-17. Line 5 selects the combination of orders to be reallocated by the mechanism based on probability. Line 6 selects the order insertion method and reallocates the orders. Line 7 computes the fitness of the new solution by using the SG technique. At line 8, the new individual is compared to the original one. If the new individual proves to be better, it replaces the original individual in line 9, and the process returns to line 5 to continue the iteration. Repeat lines 5-9 to generate and evaluate neighboring solutions for the present individual. If the newly obtained solution is superior, execute lines 5-9 for the new solution; otherwise, check at line 11 if all orders in the present solution have been checked. If there are orders in the present solution that have not been chosen to reassignment, return to line 5 to continue the iteration; otherwise, check at line 14 if the SGINO can continue its iterative solution search. If the termination condition is not met, assign τ+1 to *τ* and return to line 5 to continue the iteration; otherwise, return the current optimal individual as the best solution found using the SGINO at line 18.

**Table:** 

Algorithm 1: Pseudo-code of SGINO algorithm	Line
Initialization of algorithm parameters	1
Generating the initial solution	2
Calculating the fitness of individual solution based on SG technique	3
** While** Termination conditions are not met **do**	4
** **Select *τ* orders from the solution based on probability selection mechanism	5
** **Adaptive selection of order insertion methods to generate new individuals	6
** **Calculating the fitness of new individuals based on SG technique	7
** If** fit(new) > fit(old) **then**	8
** **Replacement of the original individual with a new one	9
** Else**	10
** If** Existing orders are not searched **then**	11
** Continue**	12
** Else**	13
** If** termination criteria not met **then**	14
** ** τ=τ+1	15
** Else**	16
** Break**	17
**Return** Final Solution	18

### 3.2. Initialization of solution individual

**Fig 1:**
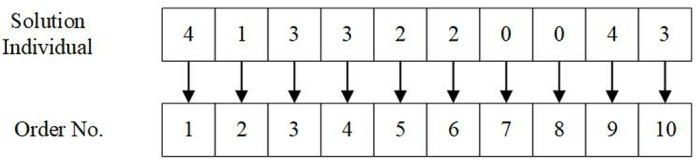
Example of solution individual.

In order to solve the stochastic order assignment issue considering carbon emissions, we construct a solution by assigning each order to an appropriate aircraft. The solution individual is composed of a sequence of elements, with the number of elements matching the count of orders that require processing. Each element value specifies the aircraft number assigned to the order. Fig 1 illustrates a solution considering 10 orders assigned to 4 airplanes. In this solution, orders 5 and 6 are assigned to aircraft 2, orders 3, 4, and 10 are to be fulfilled by aircraft 3, and orders 7 and 8 are not assigned to any aircraft, i.e., these two orders are not transported.

The initial solution generation process involves the following steps

Step 1: Arrange the orders in ascending order of delivery time into the set OS, sort the aircraft by ascending flight time into the set FS, and set the initial loading volume and weight of each aircraft to zero.

Step 2: Iterate through every order *i* in OS, and if order *i* is identified for transportation ($p_{i}=1$) or is selected for transportation ($random\left(0,1\right)<p_{i}$), assign it to the aircraft that can complete the transportation first.

Step 3: Return the resulting solution.

### 3.3. Evaluating the solution

In this paper, the solution individual is defined as a list consisting of a series of orders $x_{i}(i\in N)$, i.e., $x=\text{\{}x_{1}\text{,}...\text{,}x_{i},...,x_{n}\text{\}}$. Under a particular scenario *s*, a penalty cost is incurred if the carbon emissions from the transportation of any aircraft exceed the carbon allowance. The solution individual does not incur penalties in all scenarios, and the set of scenarios $Q_{x}$ for which it incurs penalties can be denoted as:


$$Q_{x}=\left\{s|\sum_{i\in N}\sum_{j\in M}\left(y_{i}^{s}\cdot x_{ij}\cdot t_{ij}\right)>a_{j}\right\}$$ (15)

If order *i* is not scheduled for transportation (i.e., $x_{i}=0$) in the solution , it will not increase the carbon emissions from transportation, regardless of the demand for that order *i* in the scenario. Therefore, orders that are not scheduled for transportation do not need to be considered when calculating the penalty cost of excess carbon emissions. Let ${Τ}_{x}$ denote the set of all orders in the solution that are selected for transportation, i.e., ${Τ}_{x}=\left\{i\in Ν|x_{i}=1\right\}$. $S_{x}$ denote the set of scenarios that generate penalties corresponding to $T_{x}$. Thus, the scenario $w\in S_{x}$ is described as a vector consisting of the elements $y_{i}^{w}$$\left(k\in T_{Χ}\right)$. That is, $w=\left\{y_{1}^{w},...,y_{k}^{w},...,y_{\left|T_{Χ}\right|}^{w}\right\}$. To compute the penalty cost of solution individual , only the penalty values corresponding to the scenario in the set of $S_{x}$ are considered. Thus.


$$\sum_{s\in G}\sum_{j\in M}\left(\varepsilon \cdot \pi^{s}\cdot e_{j}^{s}\right)\text{=}\sum_{s\in Q_{x}}\sum_{j\in M}\left(\varepsilon \cdot \pi^{s}\cdot e_{j}^{s}\right)\text{=}\sum_{w\in S_{x}}\sum_{j\in M}(\varepsilon \cdot \pi^{w}\cdot e_{j}^{w})$$ (16)

**Fig 2:**
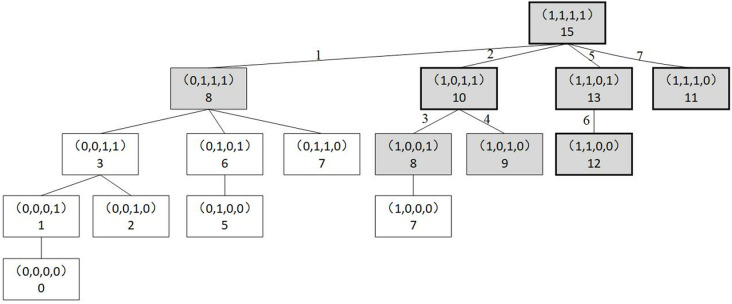
Scenario search process for generating penalties.

Fig 2 illustrates an example search process for calculating penalty costs. Assume that four orders are selected for transportation in the candidate solution, each with carbon emissions of 7, 5, 2 and 1, and the aircraft has a carbon emission limit of 9. The figure illustrates that to determine the set of scenarios that incur a penalty cost only eight scenarios need to be computed, five of which incur a penalty cost. The sum of the carbon emissions of scenario (0,1,1,1) is 8, which does not exceed the limit and therefore does not incur a penalty cost. The connecting lines between scenarios indicate the search order. Boxes with gray backgrounds indicate scenarios that have been searched and calculated, while thick borders indicate scenarios that incur penalty costs.

Algorithm 2 shows how to calculate the expected value of the carbon excess emissions penalty cost for solution . When a scenario $w^{ΙΙ}$ results from altering the *k* th item in scenario $w_{}^{Ι}$ to 0, i.e., $w^{ΙΙ}=w_{k\to 0}^{Ι}$, then $w^{ΙΙ}$ is said to be a child scenario of $w_{}^{Ι}$, and $w_{}^{Ι}$ is the parent scenario of $w^{ΙΙ}$. In the process, if *i* is smaller than $\left|T_{x}\right|$, the i+1 th element in $w_{}^{Ι}$ is changed from 1 to 0, and the resulting $w_{\left(i\text{+}1\right)\to 0}^{Ι}$ is known as the new neighboring sibling scenario of $w^{ΙΙ}$.

**Table:** 

Algorithm 2 Pseudo-code for evaluating the penalty cost for solution.	Line
**Begin** 1	1
Sequence the orders ensuring $t_{k}>t_{l}$ for every k<l 2	2
Initializing parameters: 3	3
l=1	
$w_{}^{Ι}\text{=}\left(1,1,...,1\right)$	
$\pi^{w_{}^{Ι}}\text{=}\prod_{k\in T_{x}}p_{k}$	
$P_{x}^{}=k\cdot \pi^{w_{}^{Ι}}\cdot \sum_{j\in M}e_{j}^{w_{}^{Ι}}$	
$P_{x}^{}$ =P_cost $\left(l,w_{}^{Ι}\text{,}\pi^{w_{}^{Ι}},P_{x}^{}\right)$ 4	4
**End** 5	5
**Function** P_cost $\left(l,w_{}^{Ι}\text{,}\pi^{w_{}^{Ι}},P_{x}^{}\right)$ 6	6
**For** k=l to $\left|T_{x}\right|$ **do 7**	7
$w^{ΙΙ}=w_{k\to 0}^{Ι}$ 8	8
**If** $\sum_{j\in M}e_{j}^{w^{ΙΙ}}>0$ **then** 9	9
$\pi^{w^{ΙΙ}}=\left((1-p_{k})/p_{k}\right)\pi^{w_{}^{Ι}}$ 10	10
$P_{x}^{}\text{=}P_{x}^{}\text{+}\varepsilon \cdot \pi^{w^{ΙΙ}}\cdot \sum_{j\in M}e_{j}^{w^{ΙΙ}}$ 11	11
**If** $k<\left|T_{x}\right|$ **then** 12	12
l=k+1 13	13
$P_{x}^{}$ = P_cost $\left(l,w_{}^{ΙΙ}\text{,}\pi^{w_{}^{ΙΙ}},P_{x}^{}\right)$ 14	14
**Return** $P_{x}^{}$ 15	15
**end function** 16	16

Line 2 of Algorithm 2 sorts the orders in set $T_{x}$ in descending order in terms of the carbon emissions generated by the orders. Line 3 initializes the parameters of the algorithm, where $w_{}^{Ι}$ denoting the scenario of all orders in $T_{\text{x}}$ being selected, i.e., $w_{}^{Ι}\text{=}\left(1,1,...,1\right)$, $\left(w_{}^{Ι}\in S_{x}^{}\right)$. Line 4 executes the recursive function for calculating the expected penalty cost $\sum_{w\in S_{x}}\sum_{j\in M}\left(\varepsilon \cdot \pi^{w}\cdot e_{j}^{w}\right)$. The recursive function is defined in Lines 6–14. In lines 7–9, the solution is checked in turn for the existence of a penalty cost under each child scenario of the scenario $w_{}^{Ι}$. If solution does not cause the carbon emissions of the aircraft to exceed the carbon allowance under the scenario $w^{ΙΙ}$ (i.e., $\sum_{j\in M}e_{j}^{w^{ΙΙ}}\text{=}0$), then stop the calculation of this scenario branch and continue searching for new neighboring sibling scenarios of $w^{ΙΙ}$; if solution has a penalty cost under the scenario $w^{ΙΙ}$ (i.e., $\sum_{j\in M}e_{j}^{w^{ΙΙ}}>0$), the occurrence probability of scenario $w^{ΙΙ}$ and the corresponding penalty cost for carbon excess emission are calculated in lines 10-11, and the cumulative value of the penalty cost is obtained as $P_{x}$. In line 12, the value of *k* is checked to determine whether there is a child scenario for the scenario $w^{ΙΙ}$. If $w^{ΙΙ}$ has a child scenario, i.e., *k* is smaller than $\left|T_{x}\right|$ when running $w^{ΙΙ}=w_{k\to 0}^{Ι}$, then Line 13 assigns k+1 to *l*, and the penalty value for the child scenario branch of $w^{ΙΙ}$ is computed recursively and returned at Line 14. If *k* is equal to $\left|T_{x}\right|$, $w^{ΙΙ}$ has no child and new neighboring sibling scenario, and scenario $w^{ΙΙ}$ and $w_{}^{Ι}$ stop the depth search. Line 15 indicates that the accumulated penalty cost $P_{x}$ is returned for each recursive computation. If no new scenario generates a penalty in the recursive search, the search stops and $P_{x}$ is the expected value of the penalty cost of carbon excess emissions for solution.

In the implementation of Algorithm 2, sorting orders by carbon emissions can effectively reduce the quantity of scenarios requiring calculation. Although the size of the scenarios set $S_{x}$ does not change as a result of sorting, the sorting operation reduces the number of scenarios that need to be computed. If the algorithm does not meet the stopping condition (line 9 in Algorithm 2), there is a penalty cost for solution individual in some scenarios. At this point, the recursive function executes $2^{r}$ times. The complexity of computing the solution individual is $O\left(2^{r}\right)$ for *r* binary random variables that *r* represent the uncertain number of orders.

### 3.4. Neighborhood search

#### 3.4.1. Order selection guided by probability.

In conventional ALNS algorithms, the adaptive mechanism generates new solutions through destruction and repair operations with stochastic nature. Each combination of orders that constitutes a neighboring solution is re-optimized, while some combinations may bring only limited or no improvement, which may lead to higher search costs. Typically, in the most valuable order combinations, strong orders contribute more to the objective value, while weak orders contribute less [23]. This study adopts the order selection mechanism based on probability presented by Ghoniem et al. and merges it with the ALNS algorithm to obtain an improved ALNS algorithm, which effectively improves the optimization efficiency. For each order in the solution individual, two probabilities are calculated: (1) the probability of being selected as a strong order $P_{i}^{s}=r_{i}/\sum_{i^{'}\in tempset}r_{i^{'}}$, $r_{i}$ represents the value of order *i* on the objective function; (2) the probability of being chosen as a weak order $P_{i}^{w}=\left(1-P_{i}^{s}\right)/\sum_{i^{'}\in tempset}\left(1-P_{i^{'}}^{s}\right)$. The search steps for individual neighboring solutions are as follows.

Step 1 Initialize algorithm parameters. Define the set of unsearched orders tempset for the current individual’s neighboring solution, the orders set outset that are not contained in the present individual, and the orders set subset that are selected for reassignment.

Step 2 Update the $P_{i}^{s}$ and $P_{i}^{w}$ probabilities for the unsearched orders. and alternately select strong and weak orders until the desired number of orders is reached.

Step 3 Adjusts the combination of orders based on their uncertain probabilities. Uncertain orders in outset are added to subset when the generated value falls below their selection probability. On the other hand, uncertain orders in subset are removed if the generated value exceeds their selection probability.

Step 4 Adaptively choose the insertion technique outlined in Section 3.4.2 to reassign the orders in subset.

Step 5 Clear subset and check if all orders in the present solution are searched, if not, return to Step 2.

Step 6 Outputs the neighboring solution of the individual.

#### 3.4.2. Order insertion method.

Four order insertion heuristics are used in this study: greedy insertion (GI), regret insertion (RI), greedy insertion with a noise function (GIN), and regret insertion with a noise function (RIN). They build on the existing order transportation plan by inserting selected orders into the transportation solution for each aircraft rather than completely reconstructing the solution. GI is a constructive heuristic that sequentially places the chosen orders within the primary allocation plan. (which does not contain the selected orders), choosing the point with the largest gain as the insertion location until all selected orders have been reasonably allocated. The RI method, on the other hand, inserts the orders into a sequence of orders that have already been allocated to the transportation of the aircraft, with a regret value that is the difference in the gain of the order inserted into the best location versus the second-best location. In the iteration, RI prioritizes the insertion of the aircraft with the largest regret value. GIN introduces noise on top of GI, i.e., randomly selecting insertion locations near the best insertion point. RIN introduces noise on top of RI, i.e., randomly selecting insertion points near the candidate locations after weighted selection. A more robust heuristic algorithm can be constructed by alternating between different insertion operators.

To select the insertion operators, we allocate an identical starting weight for all insertion operators and employ a roulette wheel method for selection to choose those operators that improve the solutions or increase the diversity. Following Buhrkal et al’s [26] approach, to monitor the acquired solutions, we designate a hash key for every solution and record it in a hash table. The Insertion operators’ performance is evaluated and scored throughout 100 iterations, thus ensuring that the better-performing Insertion operators have a higher probability of being selected.

### 3.5. Termination conditions

In this paper, algorithm stopping conditions are defined to end the execution of the algorithm. In the early stages of the algorithm iteration, the solution usually evolves rapidly, with better solutions appearing frequently; however, as the number of iterations increases, the frequency with which good solutions appear decreases. Given the heuristic search nature of the algorithm, it is impossible to predict if or when an best solution will be found. Therefore, deciding when the algorithm should stop running involves a balance between execution time and solution quality. In this paper, we set the stopping condition of the algorithm to terminate the algorithm if the improvement of the existing solution does not reach at least 0.25% when the neighboring size increases from *τ* to τ+1. The algorithm will return the optimal solution in the population as the best solution identified.

## 4. Numerical experiments

### 4.1. Experimental setup

The numerical experiments are based on international air cargo order data from a Chinese airline during 2018–2019, simulating real-world scenarios in air cargo order assignment. The data include transportation demands, uncertain order arrival probabilities, aircraft capacity limits, and carbon emission allowances, among other practical parameters. Experimental analyses on anonymized data validate the effectiveness and superiority of the proposed method in solving the air cargo order assignment problem. To cover the most common cases of this company, the aircraft number $m=\left\{4,5\right\}$, the order number $n\in \left\{40,60,80\right\}$, and the uncertain order number $r\in \left\{10,15,20,25\right\}$ are set. Considering the ratio of uncertain orders to the total orders in actual production, this study analyzes only the cases where the number of uncertain orders does not exceed one-third of the total orders (r≤3n).

In this study, it is assumed that the transportation cost of the order is the same for all aircraft without affecting the results of the experiment. The specific settings for the experiment are as follows: the probability $p_{i}$ for transportation is uniformly distributed as U(0,1), transportation cost coefficients $c_{ij}$ follow a uniform distribution U(10,15), transportation order revenues $v_{ij}$ follow a uniform distribution U(15,25), and transportation time coefficients $t_{ij}$ follow a uniform distribution U(8,13). In Eq. (2), the setting of the penalty coefficients *δ* and *ε* influences the results of two algorithms, but this effect is consistent for the algorithms discussed in the paper. In order to simplify the computational process, δ=1 and ε=1 are set. To evaluate the performance of the MALNS algorithm (presented in Section 3), the CGA [27] and GSPP [26] algorithms, which are applicable to the generalized assignment problem, are used for comparative analysis. Although the proposed model does not explicitly incorporate factors such as fluctuating fuel costs or regulatory constraints, these aspects can be embedded into the cost parameters or constraints of the model without altering its core structure. This flexibility ensures that the method remains adaptable to various operational scenarios.

Due to the insufficient research on stochastic optimization problems with high-dimensional uncertainty, it is not yet possible to find a performance comparison method from the existing literature that matches the intelligent stochastic optimization algorithm proposed in this study. Therefore, to demonstrate the effectiveness of the SGINO algorithm, the RSINO algorithm serves as a reference standard for performance comparison with the SGINO algorithm in Sections 4.2.1 and 4.2.2. In the RSINO algorithm, the candidate solution evaluation method of the SGINO algorithm is replaced with a random sample (RS) approach, which randomly generates transportation scenarios by simulating the selection probability of uncertain orders.

The experimental findings indicate that during the iteration of the RSINO, RS only needs to apply 1,000 scenarios for evaluation to obtain a desirable result. Therefore, in the RSINO algorithm, 1000 random scenarios will be used when evaluating the candidate solutions. Given the heuristic nature of MALNS, this study performs 20 independent runs for each algorithm in order to reduce the randomness bias of the single optimization search results. The results of these 20 runs are documented in this paper, and statistics on the mean and maximum values of the results, as well as the average solution time, are provided.

### 4.2. Experimental results

#### 4.2.1. Optimization capability analysis.

In the experimental setting described in Section 4.1, the proposed SGINO algorithm is compared and with the RSINO algorithm in terms of optimization performance. To check whether the best objective function values obtained by the SGINO and RSINO algorithms correspond to the same allocation scheme, the optimal order assignment scheme produced by the SGINO is evaluated using the RS method in the RSINO algorithm (referred to as the SGINO/RS), and the evaluation results of the candidate solution evaluation method of the SGINO are compared with the RS method for the same order allocation scheme. Tables 1 and 2 show the results of the corresponding experiments. Both the SGINO and RSINO algorithms show the average (Ave) and maximum (Max) values of the best objective function derived from the 20 experimental repetitions, while for the SGINO/RS, 1,000 and 5,000 randomized scenarios were used for the evaluation, respectively.

**Table 1:** Results of experiments on SGINO/RS, RSINO and SGINO at m = 4.

n	r	SGINO		RSINO		SGINO/RS	
		Max	Ave	Max	Ave	1000	5000
40	10	244.25	240.75	235.88	232.42	228.44	227.66
	15	209.40	200.72	207.68	198.76	210.64	209.97
	20	207.86	199.22	203.75	193.24	205.43	206.84
	25	182.72	173.08	183.10	172.20	179.82	180.24
60	10	389.18	380.68	374.64	363.27	366.60	366.83
	15	355.63	347.50	356.07	345.33	351.04	351.18
	20	336.78	313.24	337.77	311.59	335.49	336.07
	25	298.62	276.24	295.56	276.55	296.44	296.35
80	10	499.61	495.91	461.93	442.42	451.57	449.73
	15	422.68	414.65	425.55	411.12	422.81	422.24
	20	418.06	407.30	420.44	406.95	418.89	418.83
	25	421.22	403.24	419.35	402.32	420.58	419.73

**Table 2:** Results of experiments on SGINO/RS, RSINO and SGINO at m = 5.

n	r	SGINO		RSINO		SGINO/RS	
		Max	Ave	Max	Ave	1000	5000
40	10	245.08	239.98	234.01	231.27	229.80	230.01
	15	205.90	200.86	204.57	200.45	205.62	204.81
	20	206.55	196.78	202.67	193.74	204.31	205.65
	25	184.48	172.11	182.22	172.08	181.81	180.21
60	10	383.91	382.58	359.23	358.68	365.57	365.33
	15	353.34	341.83	352.62	340.97	348.77	349.00
	20	336.80	322.61	335.97	323.72	336.07	336.55
	25	289.39	274.21	290.36	274.46	287.88	287.03
80	10	497.45	490.67	455.48	446.12	446.73	445.88
	15	420.04	415.03	421.23	410.51	420.49	419.25
	20	411.55	394.90	409.32	390.39	412.77	412.78
	25	406.45	382.97	405.65	383.55	407.46	406.09

The analysis of the data in Tables 1 and 2 shows that with the number of aircraft $m=\left\{4�5\right\}$ and the number of orders $n\in \left\{\text{4}0�60,80\right\}$, if the uncertain order number r=10, most of the scenarios applied by the SGINO/RS result in the total carbon emissions of the aircraft exceeding the rated carbon emissions, making the objective function smaller than that obtained by the SGINO algorithm. When the uncertain order number $r\in \left\{15,20,25\right\}$, the results of SGINO/RS are comparable to those of the SGINO algorithm. Comparing the outcomes of SGINO and RSINO shows that incorporating SG into the intelligent algorithm can achieve comparable or superior results to RS. This indicates that the optimization performance of the SGINO exceeds that of the RSINO.

#### 4.2.2. Optimization efficiency analysis.

Table 3 presents the average computation times for the SGINO and RSINO algorithms in Section 4.2.1. To assess the optimization efficiency of these methods, the average running times of both algorithms are compared at m=4 and m=5. The data in Table 3 clearly show that the average runtime of the SGINO algorithm is considerably less than that of RSINO, which indicates that SGINO has a significant advantage in optimization efficiency.

**Table 3:** Average solution time for 20 results for SGINO and RSINO.

n	r	m = 4		m = 5	
		SGINO	RSINO	SGINO	RSINO
40	10	8.04	612.71	8.99	578.54
	15	26.73	434.87	37.19	444.62
	20	73.31	331.33	85.02	350.74
	25	188.67	298.97	152.31	345.77
60	10	10.69	918.91	12.26	904.93
	15	14.60	764.73	10.85	825.58
	20	55.57	536.95	77.09	619.46
	25	221.44	386.36	204.19	404.71
80	10	8.54	1043.29	8.22	1318.98
	15	14.63	1032.67	13.52	1178.59
	20	29.27	911.18	29.95	1246.66
	25	64.43	636.28	65.08	826.22

**Fig 3:**
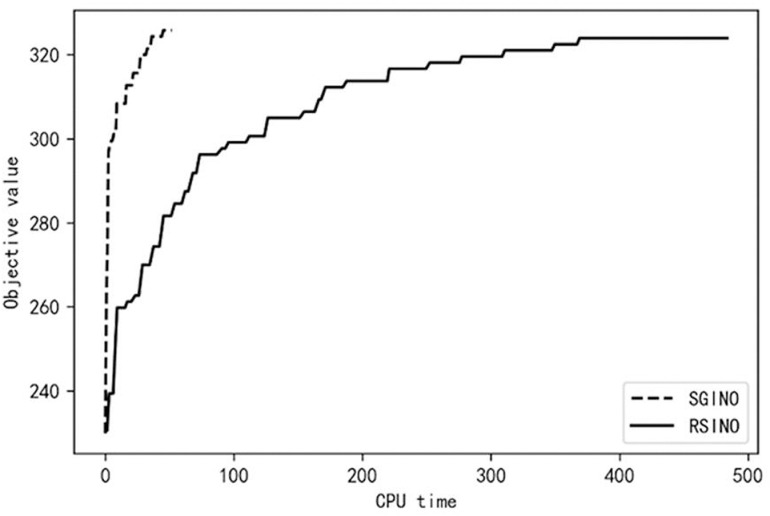
Objective values of both algorithms for m = 4.

**Fig 4:**
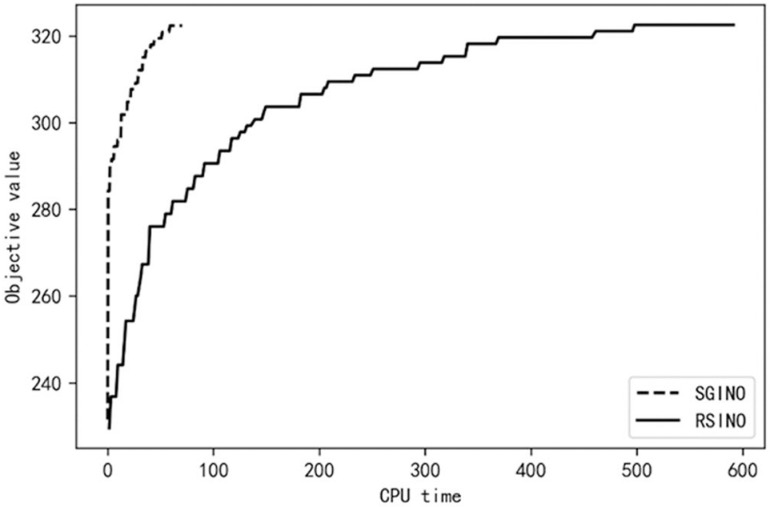
Objective values of both algorithms for m = 5.

To further analyze the performance of SGINO and RSINO algorithms, both algorithms are executed separately employing the parameter setup specified in Section 4.1. The optimization results of the algorithms SGINO and RSINO are recorded at different CPU running time *t* (unit s). Taking $m=\left\{4�5\right\}$, the order number n=60, and the uncertain order number r=20 as an example, Figs 3 and 4 show the changes in the objective values of both approaches at various times.

Figs 3 and 4 illustrates the rapid convergence of the SGINO algorithm over time. In the case of m=4, the SGINO algorithm terminates at t=51.83, where the objective function value is 325.85. The RSINO algorithm completes 300 iterations at t=483.67, and the objective function value ends up at 323.97. Below the case of m=5, the SGINO completes 300 iterations at t=70.52, where the objective function value is 322.37. The RSINO, on the other hand, reaches the objective function value of 322.56 at t=591.33. The results show that the SGINO algorithm is significantly better than the RSINO regarding optimization efficiency, and SGINO converges to a better objective function value faster than RSINO. The experimental results in other settings also show the advantage of SGINO in terms of optimization efficiency. In particular, SGINO’s superiority in efficiency is more noticeable when there are fewer uncertain orders. Since a substantial rate of order cancellations typically arises only in exceptional circumstances, the results stress the extensive adaptability and significant advantages of SGINO in high-dimensional stochastic order allocation problems considering realistic characteristics.

#### 4.2.3. Comparative performance analysis.

To validate the performance of the MALNS algorithm presented in the study, a comparative study with the GSPP and CGA algorithms is carried out. By swapping out the MALNS optimization method for GSPP and CGA in the SGINO framework, two novel algorithmic variants emerge, namely GSPP-SG and CGA-SG. Table 4 demonstrates the performance comparison of these three algorithms in several experiments. The average processing time necessary for 20 repeated executions of each algorithm is recorded in the “CPU(s)” column, while the average objective function over these 20 executions is shown in the “Ave” column.

**Table 4:** Performance comparison of SGINO algorithm with CGA-SG and GSPP-SG algorithms.

n	r	m = 4						m = 5					
		SGINO		GSPP-SG		CGA-SG		SGINO		GSPP-SG		CGA-SG	
		CPU(s)	Ave	CPU(s)	Ave	CPU(s)	Ave	CPU(s)	Ave	CPU(s)	Ave	CPU(s)	Ave
40	10	8.04	240.75	12.73	239.99	17.94	239.20	8.99	239.98	15.69	238.21	19.62	237.96
	15	26.73	200.72	38.05	199.92	51.52	199.60	37.19	200.86	64.91	200.15	82.98	200.00
	20	73.31	199.22	115.49	198.53	149.43	198.24	85.02	196.78	138.85	196.21	177.90	195.60
	25	188.67	173.08	421.78	172.65	537.67	172.30	152.31	172.11	296.66	171.41	380.91	171.25
60	10	10.69	380.68	13.51	379.88	17.13	379.44	12.26	382.58	21.81	380.76	27.27	380.54
	15	14.60	347.50	28.05	346.79	37.25	346.37	10.85	341.83	18.79	341.55	24.23	341.34
	20	55.55	313.24	82.30	312.21	106.66	311.52	77.09	322.61	146.64	320.75	190.41	319.92
	25	221.44	276.24	482.52	275.85	619.25	275.46	204.19	274.21	385.68	273.75	487.82	273.15
80	10	8.54	495.91	10.76	494.88	16.05	493.63	8.22	490.67	13.96	490.25	17.45	489.88
	15	14.63	414.65	24.12	413.55	31.83	413.39	13.52	415.03	22.24	414.16	27.78	411.68
	20	29.27	407.30	45.04	405.22	56.80	403.67	29.95	394.90	61.65	394.39	77.21	393.98
	25	64.43	403.24	100.29	400.95	127.53	400.63	65.08	382.97	115.96	381.29	145.49	380.69

The data analysis in Table 4 reveals that (1) the SGINO algorithm possesses the best optimization performance. SGINO algorithm surpasses other benchmark algorithms regarding computation time and average objective value; (2) the MALNS-based algorithms demonstrate a substantial decrease in average runtime compared to the CGA and GSPP methods. This result indicates that the MALNS optimization approach can effectively improve the performance of the SGINO algorithm, which makes the SGINO algorithm show excellent optimization ability in addressing high-dimensional stochastic order allocation problems.

The results demonstrate that the SGINO algorithm is effective in addressing the stochastic order allocation problem considering carbon emissions. The algorithm can achieve good results quickly and demonstrates strong optimization performance. In addition, the results affirm the effectiveness of the SGINO model and accentuate the significance of handling stochastic GAP problems by leveraging neighborhood search and SG methods.

#### 4.2.4. Analysis of sensitivity.

The number of scenarios, As discussed in Section 3.3, is crucial for evaluating solutions and greatly influences the algorithm’s optimization performance. During the optimization of the RSINO algorithm, increasing the number of scenarios enhances the optimization capability of the algorithm; however, decreasing the number of scenarios helps to improve the optimization efficiency. Therefore, we performed a sensitivity analysis on two main parameters related to computational complexity: the uncertain order number and the scenario number employed to assess candidate solutions in RS. The other parameters were not further analyzed in this study due to their little influence on the efficiency of optimization.

The ARD_1_ in Table 5 refers to the average relative difference (ARD) in time required for RS and SG to evaluate the same 4500 order assignment solutions. The definition of the ARD between $F^{*}$ and *F* is $ARD=\left(\left(F-F^{*}\right)/F\right)\times 100\%$. From the data analysis in Table 5, SG always outperforms RS in terms of optimization efficiency as the number of uncertain orders *r* increases.

**Table 5:** Average relative difference in time spent by RS and SG.

*n*	40				60				80			
*r*	10	15	20	25	10	15	20	25	10	15	20	25
ARD_1_	98.83%	94.73%	86.42%	67.63%	97.62%	95.36%	89.52%	74.24%	97.61%	95.74%	91.88%	85.56%

In Table 6, ARD_2_ and ARD_3_ are used to measure the average relative difference between SGINO and GSPP-SG in terms of search time and optimization results. Similarly, ARD_4_ and ARD_5_ correspond to the differences between SGINO and CGA-SG. The data in Table 6 shows that although the optimization time of SGINO increases as the parameter *r* increases, it is still significantly more efficient than the other algorithms. Especially when the number of uncertain orders reaches 25 (involving up to 2^25^ scenarios), SGINO demonstrates its superior optimization ability. This further demonstrates the robustness of the proposed approach in handling uncertain orders of various sizes.

**Table 6:** Average relative differences between SGINO, CGA-SG and GSPP-SG.

n	r	ARD_2_	ARD_3_	ARD_4_	ARD_5_
40	10	36.84%	-0.32%	55.18%	-0.65%
	15	29.75%	-0.40%	48.12%	-0.56%
	20	36.52%	-0.35%	50.94%	-0.50%
	25	55.27%	-0.25%	64.91%	-0.45%
60	10	20.87%	-0.21%	37.59%	-0.32%
	15	47.95%	-0.20%	60.81%	-0.33%
	20	32.50%	-0.33%	47.92%	-0.55%
	25	54.11%	-0.14%	64.24%	-0.28%
80	10	20.63%	-0.21%	46.79%	-0.46%
	15	39.34%	-0.27%	54.04%	-0.30%
	20	35.01%	-0.52%	48.47%	-0.90%
	25	35.76%	-0.57%	49.48%	-0.65%

#### 4.2.5. Management insights.

The management revelations of this paper include the following specifics. First, the SG technique provides researchers with an effective tool for dealing with real-life complex problems involving random variables. Compared with traditional random sampling methods, the SG technique is more efficient in dealing with high-dimensional random variable problems, which has been confirmed by experiments. Second, business managers can adopt this method to deal with stochastic order allocation problems that consider realistic characteristics. The methodology demonstrates excellent optimization efficiency while ensuring solution quality. Computational experiments also demonstrate the method’s superiority over other comparison approaches. Third, the high-dimensional stochastic assignment problem considering carbon emissions explored in this study exemplifies a stochastic combinatorial optimization problem. The real world presents many analogous stochastic problems, such as the existence of stochastic edges in network design problems, customer uncertainty in vehicle road problems, and capacity constraints in network flow problems. The developed intelligent stochastic optimization methods can not only be directly applied to the issues but can also be generalized to efficiently solve other similar problems. Overall, these insights offer guidance for designing methods to address stochastic combinatorial optimization problems and inform the practical application of the framework.

## 5. Conclusion

This study investigates the air cargo transportation order assignment problem under high-dimensional uncertainty, considering the impact of carbon emissions, with the objective of maximizing the expected transportation profit. An intelligent stochastic optimization method is developed by incorporating the scenario generation technique. The method utilizes an improved adaptive large-neighborhood search algorithm to find the optimal solution and evaluates the solution through the scenario generation technique. The proposed method is validated by a series of numerical experiments, and its efficiency exceeds that of other comparative algorithms while ensuring the optimization performance. Research has shown that taking order uncertainty into account helps companies to develop more realistic order-processing solutions under complex market environments and strict environmental requirements. This not only helps to weigh the processing cost against the penalty cost but also develops a more effective order allocation strategy.

Nonetheless, this study has certain limitations. The method primarily focuses on the efficiency and effectiveness of solving high-dimensional stochastic optimization problems and does not thoroughly examine its performance in multi-objective scenarios or under more complex constraints.

Future research could enhance the current approach by exploring more advanced stochastic variable modeling techniques, such as incorporating fuzzy variables or addressing dynamic environmental stochastic changes. Additionally, the method holds potential for application in other classic stochastic combinatorial optimization problems, such as network design and vehicle routing. Expanding the research to include multi-objective optimization and investigating real-time applications in dynamic, practical environments are also promising directions. Furthermore, future studies could integrate broader sustainability dimensions—such as noise pollution and lifecycle environmental impacts—into the optimization framework, providing a more comprehensive and sustainable solution for air cargo operations.
